# The effect of verbalization strategy on wisconsin card sorting test performance in schizophrenic patients receiving classical or atypical antipsychotics

**DOI:** 10.1186/1471-244X-6-3

**Published:** 2006-01-26

**Authors:** Alessandro Rossi, Enrico Daneluzzo, Annarita Tomassini, Francesca Struglia, Roberto Cavallaro, Enrico Smeraldi, Paolo Stratta

**Affiliations:** 1 Department of Experimental Medicine, University of L'Aquila, Italy; 2 Department of Neuroscience, Libera Università "Vita Salute S.Raffaele" Milano, Italy; 3 Department of Mental Health, A.U.S.L. 4 L'Aquila, Italy

## Abstract

**Background:**

A number of reports showed en encouraging remediation in some patients' executive deficits thanks to the use of 'information processing strategies'. Moreover the impact of antipsychotics on cognitive functions of the schizophrenics is an important issue, especially if an integrated psychosocial treatment is needed.

The aim of this paper is to evaluate different executive performance and response to verbalization, a strategy of the Wisconsin Card Sorting Test (WCST) remediation, in subjects on classical vs atypical antipsychotic (AP) treatment.

**Methods:**

Sixty-three schizophrenic subjects undertook the WCST under standard and modified (verbalization) administration. Subjects were stratified by the kind of WCST response (i.e. good, poor and remediable) and AP treatment (i.e. atypical vs. classical).

**Results:**

Subjects on atypical APs showed a better performance than those on classical ones. More poor performers who did not remediate were seen in the sample with classical Aps while subjects who remediated the performance were seen in the subgroup with atypical APs only. An increase of perseverative and total errors was seen in poor performers subjects on classical APs.

**Conclusion:**

Subjects on atypicals showed a better cognitive pattern in terms of WCST performance. Since the naturalistic assignment of medication we cannot draw conclusions about its effect on cognitive performance and its interaction with cognitive remediation potential. However the data lead us to hypothesize that subjects with potential room for remediation did so with the atypical APs.

## Background

Cognitive deficits are important targets for intervention in patients with schizophrenia to favor clinical and functional success. Executive skills are relevant for dealing with novel and complex situations, crucial for occupational outcome and independent living of people with schizophrenia [1,2]. Several studies reported that a large part of these people improve their executive performance by remediation strategies and cognitive therapies [3-5].

A number of reports showed en encouraging remediation in some patients' executive deficits thanks to the use of 'information processing strategies'. This has been mostly investigated using Wisconsin Card Sorting Test (WCST), and verbalization has been found the most efficacious procedure to obtain the performance improvement. Cumulatively, these data suggest that a simple instruction may enhance executive function and affect WCST performance in patients with schizophrenia [6-9].

In our previous studies on remediation of WCST performance of persons with schizophrenia we hypothesised that the improvement could be attained through a cognitive strategy that bounds schizophrenic patients to use a conceptually driven processing. Using verbalization as such a strategy, we observed that when the patients were required to verbally express the matching criterion before the card sorting, a remediation of the poor WCST response occurred [10,11]. Furthermore, we observed different patterns of response to verbalization, i.e. improvement or worsening of the performance. About the 63% of the WCST poor performers improved while the remaining patients, showed an increase of perseverative errors. The latter subjects were characterized by more negative symptoms, poor outcome and earlier age at onset. On the basis of these observations we hypothesized that this modified WCST administration could be used as a criterion that, if validated, could be able to unveil different patient subgroup with different pathophysiological trajectories.

The impact of antipsychotics (APs) on cognitive functions of the schizophrenics is an important issue, especially if an integrated psychosocial treatment is needed [2]. Even though cognitive enhancement in schizophrenia is far from being a resolved issue [12], there is evidence that atypical APs can provide some cognitive benefit (for reviews see [1,13]]. However, caution is needed before generalize the results of these studies because the limited effect size and deficient methodologies [13].

The aim of this study is to compare the cognitive performance and response to verbalization in subjects on classical vs atypical APs.

## Methods

### Subjects

A sample of 63 consecutive out-patients (18 females and 45 males) who met the DSMIII-R criteria for schizophrenia were evaluated cross-sectionally along a naturalistic perspective. Diagnoses were made by a senior psychiatrist (P.S.) who personally interviewed the patients according to the Structured Clinical Interview for DSM-III-R [14]. The subjects were excluded if had history of head injury, alcohol abuse or serious neurological or physical disease. None of the patients had been hospitalized in the past six months and all were relapsing multiepisode patients able to live in the community on maintenance AP therapy. The mean age was 32.60 years (SD 7.48), and educational level (number of successfully achieved classes) was 11.12 years (SD 2.85). Age at onset of symptoms was 23.00 ± 5.07 and length of illness 10.00 ± 6.65 years. All were taking APs, and the mean chlorpromazine-equivalent dose [15,16] was 403 (SD 195.09) at the time of the evaluation.

In order to avoid variables that could reduce the power of our investigation only patients on atypical or classical AP mono-therapy, clinically stabilized and well functioning in the community were selected. Patients were considered clinically stabilized if no single item of the Positive and Negative Syndrome Scale (PANSS) [17] exceeded the definition 'mild' of the symptom (no more than 3 in a range 1–7). Good functionality was considered satisfied if the subject reported 65 or more on the Global Assessment of Functioning Scale (G.A.F., D.S.M. – IV) in the past 6 months.

All participants provided written informed consent after complete description of the study, in accordance with the local university institutional review board (Ethic Committee A.U.S.L. 4 L'Aquila)

### Procedure

All subjects undertook two separate WCST sessions (128 cards). The first administration was along the standard instructions as described by Heaton [18]; at the second test administration, one hour after the first one, the subjects were also required to 'verbally express' the matching criterion before the card sorting. The performance was considered poor or successful on the basis of the number of categories achieved: poor if less o equal 3, successful if 4 or more according to previous studies [18,6,10]. Three possible kinds of performance were considered: a) good performance if the subject achieved 4 or more categories both at the first and second administration; b) remediable performance if the subject reported a poor performance at the first but good at the second one; c) poor performance at both the administrations.

WCST indexes used in the subsequent calculations are the number of completed categories (CC), perseverative errors (PE), total errors (TE) and unique errors (UR).

### Statistical analysis

Two-way ANOVA with remediation effect (standard administration vs. verbalization) as within subjects factor and atypical or classical AP therapy, as between subjects factor, was performed separately on the WCST indexes as dependent variables. Chi square and Student t-test were used when necessary. All analyses yielding a p value of less than 0.05 were considered significant.

## Results

Forty of the 63 subjects (63.5%) were good performers and 23 poor performers; of these latter 7 were the subjects who remediated (the 30.4% of the poor performers at standard WCST administration).

WCST performance and distribution of the total sample along the 'remediation pattern' (Good, Remediable and Poor) is reported in the Table 1. Both Good and Remediating subjects improved their performance while Poor performers at both WCST administrations worsened in terms of an increase of perseverative and total errors.

**Table 1:** Distribution and WCST performance under standard or modified administration (verbalization) of the studied sample.

	WCST Performance			
	
WCST indexes	Total sample (n = 63)	Good (n = 40)	Poor (n = 16)	Remediable (n = 7)
Categories Achieved				
Standard adm.	3.9 ± 2.4#	5.6 ± 0.9	0.6 ± 0.9*	2.0 ± 1.4§
Verbalization	4.6 ± 2.0	5.8 ± 0.4	1.3 ± 0.9	5.4 ± 0.7
Perseverative Errors				
Standard adm.	17.4 ± 11.9*	14.4 ± 11.2#	23.8 ± 11.6**	20.0 ± 12.0
Verbalization	13.0 ± 17.6	4.8 ± 9.2	34.2 ± 19.0	11.0 ± 9.5
Total Errors				
Standard adm.	30.9 ± 15.8§	23.7 ± 13.8#	43.6 ± 10.8§	42.8 ± 11.1*
Verbalization	23.6 ± 23.6	11.6 ± 15.6	53.4 ± 16.0	23.9 ± 15.1
Unique Errors				
Standard adm.	1.9 ± 4.4*	1.1 ± 2.2*	1.8 ± 3.1	6.7 ± 10.6
Verbalization	0.8 ± 3.0	0.1 ± 0.4	1.6 ± 5.0	2.8 ± 4.5

Paired t-tests Standard adm vs Verbalization: * *P *< 0.05; ** *P *< 0.01; § *P *< 0.005; # *P *< 0.0005

33 (52.4%) patients were taking atypical Aps. More males than females were taking classical APs (83.3% of subjects with classical AP treatment vs. 60.6% of subjects on atypicals; Chi-Square = 3.97, d.f. = 1, *P *< 0.05). No differences were seen in total PANSS score between the 2 groups (classical APs 72.6 ± 22.7, atypical 76.00 ± 18.90).

When further stratification was made on the basis of atypical or classical AP therapy, more poor performers not remediating were seen in the sample with classical APs (68.75% of the poor performers) while remediators were only present in the subgroup with atypical APs (Chi-Square = 9.28, d.f. = 2, *P *< 0.01 for 2 × 3 contingency table). The WCST performances stratified by kind of response to the standard and modified WCST administration and AP treatment are reported in the Table 2.

**Table 2:** Distribution and WCST performance under standard or modified administration (verbalization) of subjects on atypical and classical AP treatment.

WCST indexes	WCST performance						
	
	Total sample		Good		Poor		Remediable
				
	Atypical AP (n = 33)	Classical AP (n = 30)	Atypical AP (n = 21)	Classical AP (n = 19)	Atypical AP (n = 5)	Classical AP (n = 11)	Atypical AP (n = 7)
Categories Achieved ^a, e, i^							
Standard adm.	4.1 ± 2.4§	3.8 ± 2.4*	5.8 ± 0.5	5.4 ± 1.2	0.0 ± 0.0	1.0 ± 1.0	2.0 ± 1.4§
Verbalization	5.0 ± 1.9	4.2 ± 2.1	5.9 ± 0.4	5.8 ± 0.5	0.8 ± 1.3	1.5 ± 0.6	5.4 ± 0.7
Perseverative Errors ^b, f, l^							
Standard adm.	13.8 ± 9.0	21.4 ± 13.5	10.5 ± 6.6#	18.7 ± 13.6§	19.2 ± 7.8	26.0 ± 12.7§	20.0 ± 12.0
Verbalization	6.9 ± 8.7	19.6 ± 22.2	2.7 ± 5.0	7.1 ± 12.1	18.8 ± 6.5	41.3 ± 18.7	11.0 ± 9.5
Total Errors ^c, g, m^							
Standard adm.	27.1 ± 13.9#	35.0 ± 17.0	19.1 ± 8.3#	28.8 ± 16.9**	38.8 ± 8.9	45.8 ± 11.2*	42.8 ± 11.1*
Verbalization	17.3 ± 17.6	30.5 ± 27.5	8.1 ± 9.7	15.4 ± 19.8	46.6 ± 10.0	56.6 ± 17.6	23.9 ± 15.1
Unique Errors ^d, h, n^							
Standard adm.	2.3 ± 5.6	1.5 ± 2.4§	1.0 ± 2.4	1.2 ± 1.9*	1.6 ± 3.5	1.9 ± 3.1	6.7 ± 10.6
Verbalization	1.5 ± 4.1	0.1 ± 0.3	0.2 ± 0.6	0.0 ± 0.2	5.0 ± 8.6	0.1 ± 0.5	2.8 ± 4.5

2-way mixed ANOVAs

Total sample

AP treatment effect (A): ^a ^F = 1.0 NS; ^b ^F = 10.7 *P *< 0.0025; ^c ^F = 5.7 *P *< 0.025; ^d ^F = 1.8 NS.

Remediation effect (B): ^a ^F = 14.6 *P *< 0.0005; ^b ^F = 5.6 *P *< 0.025; ^c ^F = 10.3 *P *< 0.0025; ^d ^F = 5.6 *P *< 0.025.

A × B interaction: ^a ^F = 1.51 NS; ^b ^F = 2.04 NS; ^c ^F = 1.43 NS; ^d ^F = 0.47 NS

Good performers

AP treatment effect (A): ^e ^F = 1.5 NS; ^f ^F = 5.5 *P *< 0.025; ^g ^F = 4.8 *P *< 0.05; ^h ^F = 0.0 NS.

Remediation effect (B): ^e ^F = 2.9 NS; ^f ^F = 35.1 *P *< 0.0005; ^g ^F = 26.4 *P *< 0.0005; ^h ^F = 7.0 *P *< 0.025.

A × B interaction: ^e ^F = 1.1 NS; ^f ^F = 1.3 NS; ^g ^F = 0.2 NS; ^h ^F = 0.4 NS

Poor performers

AP treatment effect (A): ^i ^F = 4.9 p < 0.05; ^l ^F = 4.6 *P *< 0.05; ^m ^F = 1.7 NS; ^n ^F = 1.4 NS.

Remediation effect (B): ^i ^F = 6.4 *P *< 0.025; ^l ^F = 4.9 *P *< 0.05; ^m ^F = 7.6 *P *< 0.025; ^n ^F = 0.6 NS.

A × B interaction: ^i ^F = 0.2 NS; ^l ^F = 5.5 *P *< 0.05; ^m ^F = 0.2 NS; ^n ^F = 6.0 *P *< 0.05

Paired t-tests Standard adm vs Verbalization: * *P *< 0.05; ** p < 0.01; § *P *< 0.005; # *P *< 0.0005

In the total sample, subjects with atypical APs showed less perseverative and total errors than those on classical, both at standard or modified administration; moreover subjects assuming atypical APs only, significantly reduced perseverative and total errors with verbalization.

When the kind of response to the modified administration was examined, an increase of perseverative and total errors in poor performers was seen among the subjects on classical APs only (interaction F = 5.50, *P *< 0.05). A similar interaction, without treatment or verbalization effect, with an increase of unique errors in subjects on atypicals was also seen.

## Discussion

Subjects on atypical AP treatment reported a better executive function pattern in terms of WCST performance than those on classical one. Moreover with the modified WCST administration, remediation does occur in the atypical group only, while poor performers on classical Aps did worsen their performance.

Our observation is in agreement with the results from Reeder et al. [19] study on executive skills reporting that people on atypical AP medication show greater improvement in working memory than those on classical; the greatest benefit was gained by those who both received Cognitive Remediation Therapy (CRT) and were prescribed atypical antipsychotic medication. On the other hand cognitive and psychosocial improvement from Cognitive Enhancement Therapy (CET) was found unrelated to the type of AP medication received [20]. This study was not however focused on executive functions evaluation but neuropsychological composites and cognitive style index are reported. Similarly in a randomized controlled study of the effects of a cognitive remediation program on adolescents with early onset schizophrenia, no significant differences were found between the subjects on classical and atypical Aps [21].

Moreover we replicated and expanded our previous observations [10,11] in a new larger independent sample, although the percentage of subjects with good or remediating performance is remarkably different (good performers: present study 63.5%, previous study 26.9%; remediating subjects: present study 30.4%, previous study 63.2%) [11]. This could be due to the selection bias, because we studied subjects with good functionality only.

The naturalistic assignment of medication does not permit to draw definitive conclusions about the effect of medication on cognitive performance and its interaction with cognitive remediation potential. Because of the design of the study we do not state a priori explicit and research criteria for AP treatment assignments. This decision has been left on clinical judgment. We cross-sectionally studied and compared the two groups.

However medication type might be not strictly correlated with clinical features of the disorder (i.e. development of the illness, treatment failures, patient's cognitive profile etc.): it could be possible that patients with more recent onset have been treated with atypical APs because these were more widely available, while older clinically stable patients remained on classical APs. In other words, criteria leading to the AP treatment choice could be influenced by socioeconomical and historical factors too. These factors are well beyond the scope of this article.

Another potential limitation could be due to fixed order WCST administrations: the remediation could reflect merely practice effect instead of an effect of verbalization. However there is a dearth of literature on cognitive test practice effects in schizophrenia, likely because of the scarce possibility of improvement [22]. One study [23] reported no evidence of practice effect, repeating executive tests after 3 weeks, in stable treatment-resistant patients treated with clozapine. Stratta et al. [10] administering four times WCST in two days did not observe improvement in patients on classical AP medication. Harvey et al. [24] found that the degree of improvement with practice was greater in patients administered an atypical medication than in those given typical medication. However the possibility to achieve practice effect, previously limited by classical AP treatment, could also been an important result. The restoration of a practice effect is real advantage although does not represent actual procognitive effectiveness [25,26]. Follow up- studies are needed to better clarify the meaning of the 'remediation'.

## Conclusion

Although we are still far from the point of a meaningful integration of cognitive findings with AP treatment outcome, results from neuropsychological assessment could provide guidance to the patient's management. We could hypothesize that patients who do not remediate, even if in a state of clinical response to classical AP treatment, could have a trial with atypicals to evaluate a chance of cognitive improvement. On the other hand subjects who do not show improvement could benefit from a different attentional training using rehabilitation methodologies more weighted on procedural learning or practice effect [27].

Improvement in cognitive functioning is one of the most important clinical targets in the treatment of schizophrenia [28]. In the cognitive 'puzzle', we propose that the remediation 'piece' is associated with better cognitive functioning and atypical AP treatment. We need further study to evaluate how these three variables are connected and what kind of clinical predictions they offer.

## Authors' contributions

AR and ES conceived and designed the study, were involved in the revisions. AT, FS and RC collected neurocognitive test data and co wrote first draft. ED and PS advised on statistical analysis, helped to write the corresponding sections and drafted the manuscript. All authors read and approved the final manuscript.

## Pre-publication history

The pre-publication history for this paper can be accessed here:


